# Intra-articular delivery of celastrol by hollow mesoporous silica nanoparticles for pH-sensitive anti-inflammatory therapy against knee osteoarthritis

**DOI:** 10.1186/s12951-020-00651-0

**Published:** 2020-07-08

**Authors:** Tian Jin, Di Wu, Xiao-Ming Liu, Jiang-Tao Xu, Bing-Jie Ma, Yun Ji, Yu-Ying Jin, Si-Yin Wu, Tao Wu, Ke Ma

**Affiliations:** 1 Department of Pain Medicine, Xinhua Hospital Affiliated to Shanghai Jiao Tong University School of Medicine, Shanghai, 200092 China; 2 Department of Urology, Xinhua Hospital Affiliated to Shanghai Jiao Tong University School of Medicine, Shanghai, 20092 China; 3 Shanghai Jiao Tong University School of Medicine, Shanghai, 200025 China

**Keywords:** Celastrol, Hollow mesoporous silica nanoparticle, Osteoarthritis, Intra-articular injection, pH-responsive

## Abstract

**Background:**

Celastrol has been proven effective in anti-inflammatory but was limited in the clinic due to the poor solubility and side effects induced by low bioavailability. Osteoarthritis has acidic and inflammatory environment. Our aim was to load celastrol into HMSNs and capped with chitosan to construct a pH-responsive nanoparticle medicine (CSL@HMSNs-Cs), which is of high solubility for osteoarthritis intra-articular injection treatment.

**Methods:**

The CSL@HMSNs-Cs were assembled and the characteristics were measured. The CSL@HMSNs-Cs was applied in vitro in the chondrocytes collected from rats cartilage tissue and in vivo in the MIA induced knee osteoarthritis rats via intra-articular injection. Cytotoxicity assay, pH-responsive release, pain behavior, MRI, safranin o fast green staining, ELISA and western blot analysis were applied to evaluate the bioavailability and therapeutic effect of CSL@HMSNs-Cs.

**Results:**

CSL@HMSNs-Cs was stable due to the protection of the chitosan layers in alkaline environment (pH = 7.7) but revealed good solubility and therapeutic effect in acidic environment (pH = 6.0). The cytotoxicity assay showed no cytotoxicity at relatively low concentration (200 μg/mL) and the cell viability of chondrocytes stimulated by IL-1β was increased in CSL@HMSNs-Cs group. Paw withdrawal threshold in CSL@HMSNs-Cs group is increased, and MRI and Safranin O Fast Green staining showed improvements in articular surface erosion and joint effusion. The upregulated expression levels of IL-1β, TNF-α, IL-6, MMP-3 and MMP-13 and NF-κB signaling pathway of chondrocytes were inhibited in CSL@HMSNs-Cs group.

**Conclusion:**

Hollow mesoporous silica nanoparticles were an ideal carrier for natural drugs with poor solubility and were of high biocompatibility for intra-articular injection. These intra-articular injectable CSL@HMSNs-Cs with improved solubility, present a pH-responsive therapeutic strategy against osteoarthritis.

## Introduction

Osteoarthritis (OA) is a prevalent joint disease worldwide characterized by the progressive destruction of articular cartilage, joint inflammation, osteophytes, subchondral bone remodeling, joint effusion and pain [1]. The incidence of OA has significantly increased due to the aging population and the growing number of obese individuals [2, 3]. OA is a leading cause of disability, and knee pain is the major reason for repeated medical visits [4]. The mechanisms of OA pain are complex and involve abnormalities of both the peripheral and central nervous systems [5]. Continuing nociceptive stimulation from inflammation in joints lowers the central sensitization [6].

The pathogenesis of OA involves disruption of the delicate balance between repair and destruction of joint tissues due to mechanical stress and inflammatory cytokines [7]. Efforts in targeted toward knee osteoarthritis (KOA) therapeutic strategies have largely focused on systemic drug interventions, the mainstay of which is the long-term use of nonsteroidal anti-inflammatory drugs [8]. But systemic drug interventions have also been reported to be related to a high incidence of cardiovascular events and gastrointestinal side effects [9]. Arthroscopic knee surgery has little to no effect on pain relief, and the benefits are limited in time accompanied by adverse events such as deep venous thrombosis [10].

Intra-articular injection is a local drug application without the risk of systemic side effects and has been preferred in the management of KOA due to the advantages of drug localization, minimal invasiveness, rapid onset and significant pain relief. Current existing therapeutic agents are glucocorticoids, such as betamethasone and triamcinolone acetonide. However, long-term use of intra-articular injection of glucocorticoids could induce marked destructive cartilage changes [11].

All these facts have motivated the search for more efficacious drugs with fewer side effects.

Celastrol (CSL), a pentacyclic triterpene extracted from the roots of *Tripterygium wilfordii*, has been widely used for the treatment of rheumatoid arthritis, systemic lupus erythematosus and cancers [12, 13]. Previous related studies have shown that IL-1β and TNF secretion can be blocked by celastrol in OA animals due to its effect on the abolishment of immune cellular infiltration and proliferation, preventing cartilage and bone damage [14]. However, poor bioavailability owing to its poor water solubility (water solubility: 13.25 ± 0.83 μg/mL at 37 °C) and systemic toxicity limits its clinical therapeutic properties [15].

Hollow mesoporous silica nanoparticles (HMSNs) have proven to be promising nanoparticles in nanomedicine fields due to their advantages of great loading capability, multitudinous functionalization and good biocompatibility [16]. HMSNs have shown great potential in terms of cargo loading capability and solubility because of their large specific surface area and pore volume. Under normal physiological conditions, the pH of synovial fluid is usually alkali (pH 7.7); however, the pH of OA synovial fluid can decrease to pH 6.0 due to the deposits of inflammatory metabolic products in and around joint tissue [17]. Chitosan is an amino-polysaccharide with advantages of biocompatibility and natural nontoxicity. Chitosan is often chosen as a gatekeeper due to its pH responsiveness and control the release of cargo drugs of nanoparticles in different pH environments [18]. The pH-sensitive nanocomplex that diffuses in the joint cavity is concentrated released in the areas with severe inflammation, which also means the chondrocytes in the acidic microenvironment. Thereby reducing the risk of adverse reactions caused by overdose.

All these characteristics make HMSNs an appealing drug delivery carrier for celastrol to construct a new pH-responsive nanomedicine to inject via intra-articular. In this study, we assembled celastrol into HMSNs, utilizing chitosan (Cs) as a coating to construct a nanomedicine (CSL@HMSNs-Cs, CHC) with high delivery efficiency. Our aim was to observe the effect of CSL@HMSNs-CS on OA treatment and regulation of the NF-κB signal pathway to find novel therapies for OA and provide a reference for the application of poorly soluble drugs.

## Result and discussion

### Synthesis and characterization of CSL@HMSNs-Cs

**Scheme 1:**
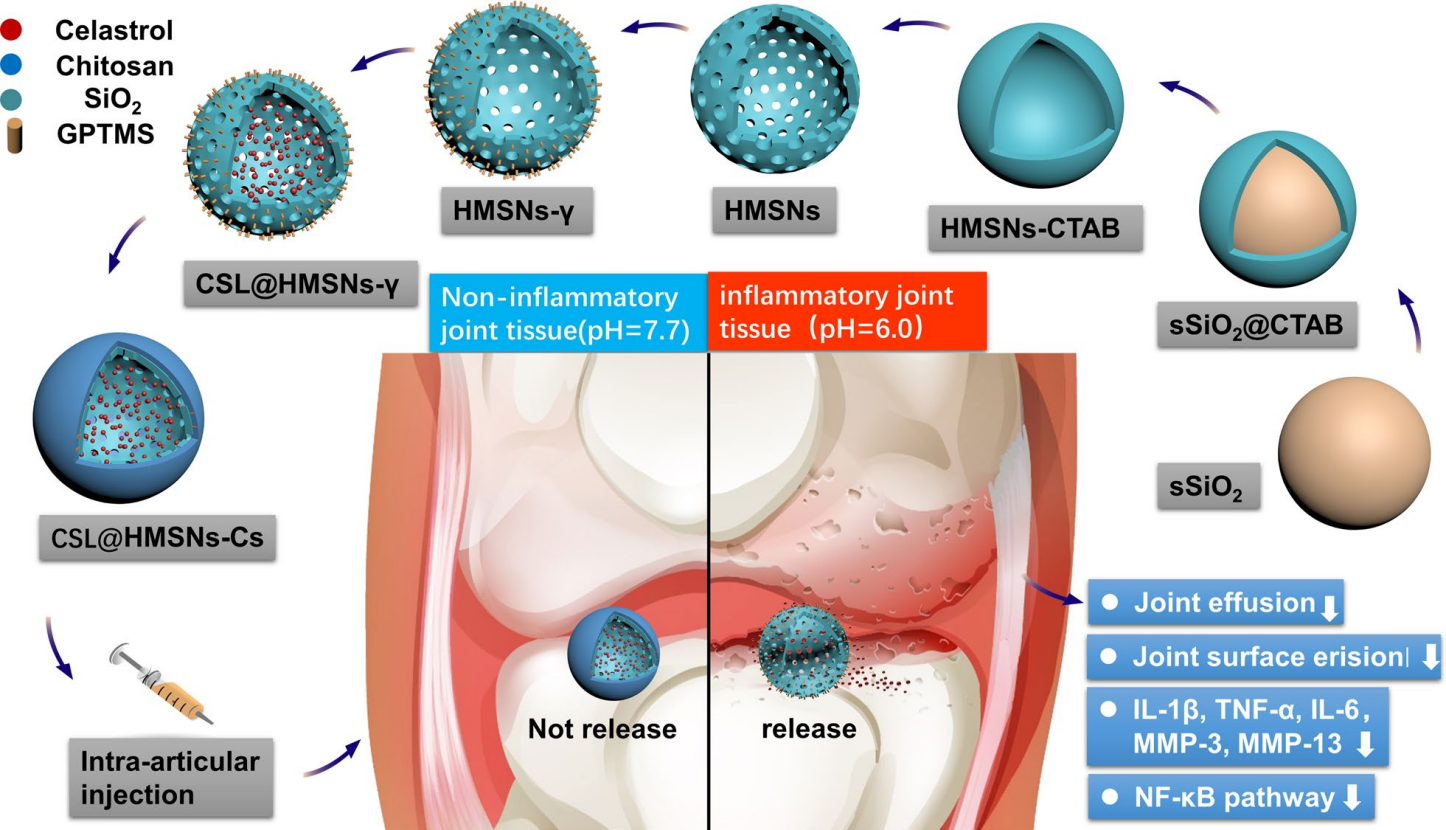
Schematic assembly procedure of CSL@HMSNs-Cs and mechanism of intra-articular injection of CSL@HMSNs-Cs serving as a pH-responsive medicine for anti-inflammatory therapy of osteoarthritis

The preparation protocol for CSL@HMSNs-CS is shown in Scheme 1. Solid silica nanoparticles (sSiO_2_) were first synthesized as a core via a modified Stöber method [19] and a mesoporous silica shell structure was coated with TEOS using the surfactant CTAB as a stabilizer. The core was selectively etched out by Na_2_CO_3_ aqueous solution at a temperature of 80 °C to form a hollow structure. The CTAB was removed by hydrochloric acid–ethanol solution and the HMSNs were collected. The diameter of the hollow core depended on the size of sSiO_2_, which depended on the ratio of reagents and reaction time of the sSiO_2_ synthesis process [19]. Then CSL was loaded in the HMSNs by free diffusion. Finally, GPTMS, which contains both epoxide ring reactive to amino groups of chitosan and also hydrolyzable methoxysilyl groups, reactive to hydroxyl groups of HMSNs, was chosen as a crosslinking agent to anchor Cs onto HMSNs.

**Fig. 1:**
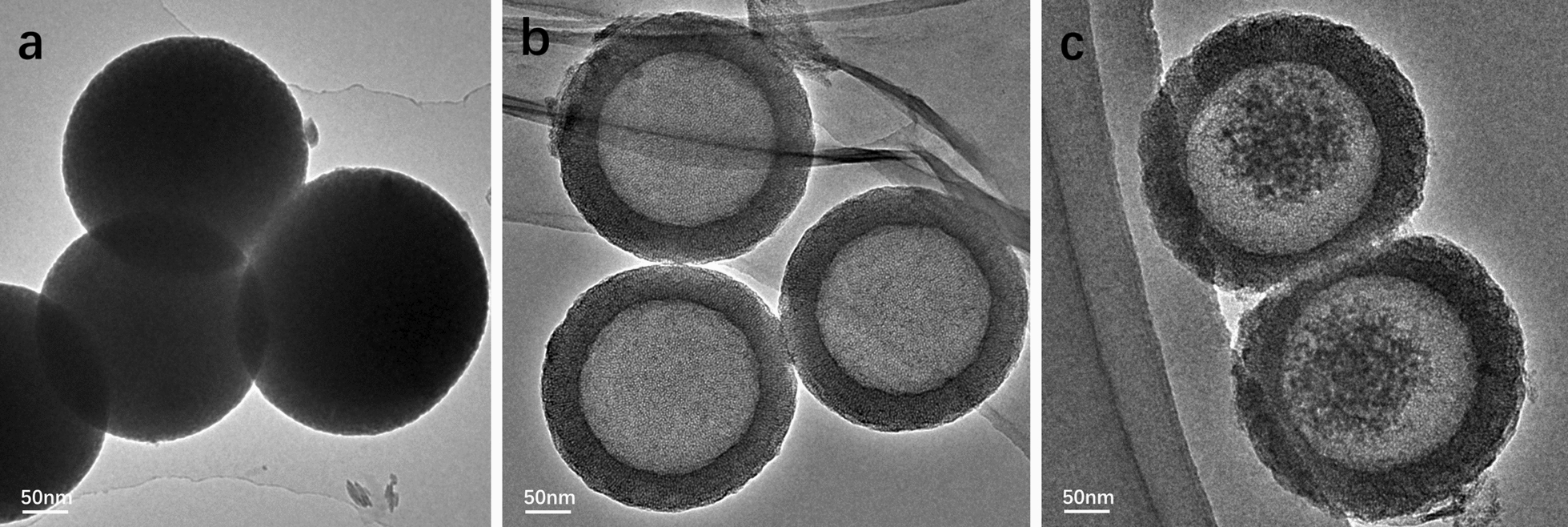
Transmission electron microscopy (TEM) images of solid SiO_2_ (**a**), HMSNs (**b**) and CSL@HMSNs-Cs (**c**). Scale bars, 50 nm

The morphologies of HMSNs and CSL@HMSNs-CS were observed by transmission electron microscopy (TEM). In Fig. 1, the images show a solid sphere (Fig. 1a) and a homogeneous spherical shape (size: ~ 260 nm) with uniform large hollow cavities (size: ~ 170 nm) (Fig. 1b). After loaded with CSL and modification of Cs, the shell was thicker and obscured and CSL was observed in the cavity (Fig. 1c).

**Fig. 2:**
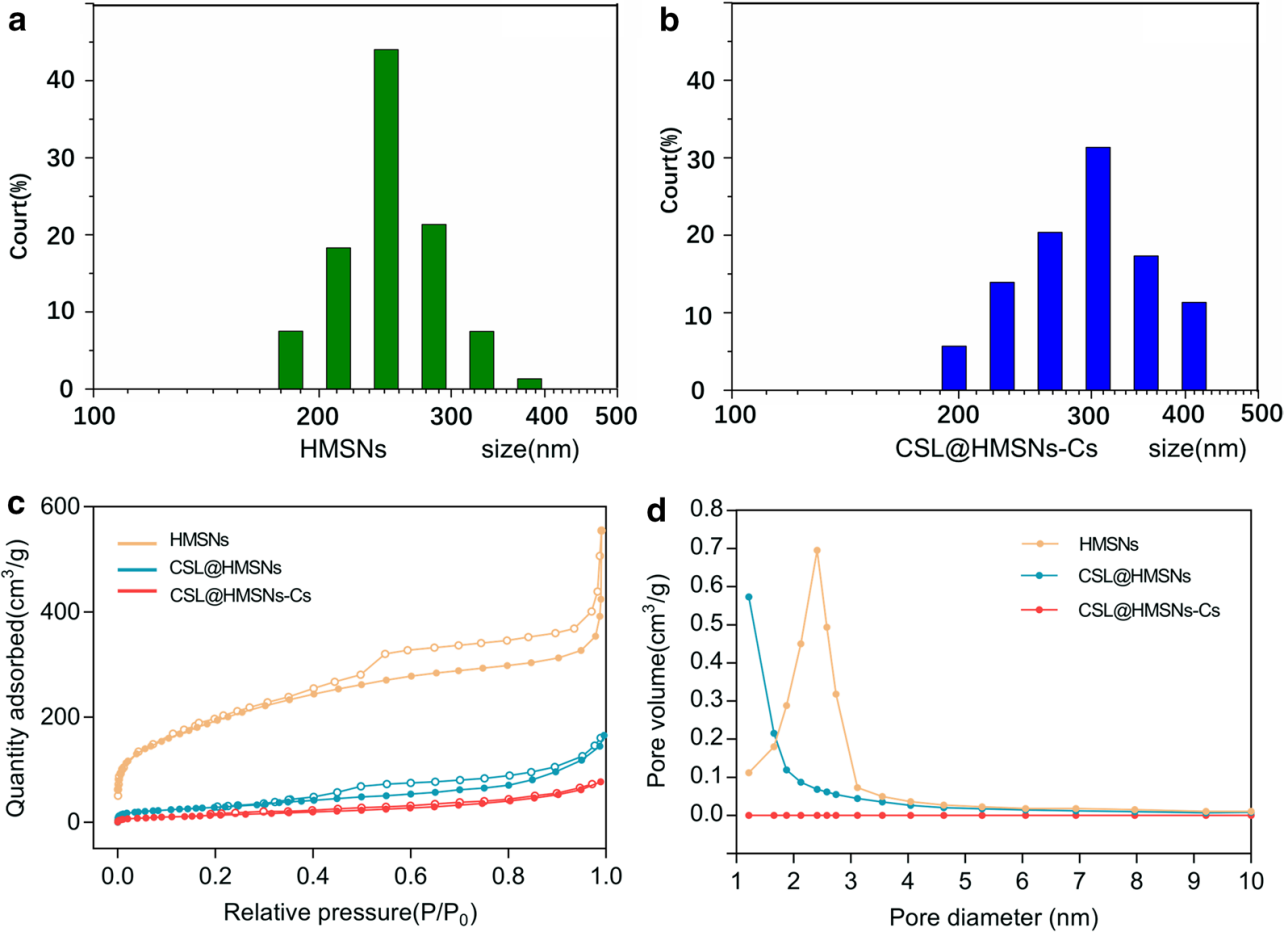
Characterization of HMSNs, CSL@HMSNs and CSL@HMSNs-CS. The dynamic light scattering (DLS) particle size distributions (**a**, **b**), N_2_ adsorption–desorption isotherms (**c**) and pore size distribution curves (**d**) of nanoparticles

The dynamic light scattering (DLS) particle size distribution results in Fig. 2a, b showed a slight increase after coating with Cs (average 260.76 nm to 290.17 nm).

The N_2_ adsorption–desorption isotherm curves further manifest a classical Langmuir type IV isotherm with a type H_2_ hysteresis loop, indicating the existence of mesoporous structures of HMSNs (Fig. 2c). The mesopores structure was obscured after surface modification and showed a significant pore-coating effect by Cs.

The BET pore diameter of the HMSNs was determined to be approximately 2.4 nm (Fig. 2d) by nitrogen adsorption investigation, while the pore volume (V_pore_) and specific surface area (S_BET_) were 0.7668 cm^3^/g and 1006.8 m^2^/g, respectively. The V_pore_ and S_BET_ of CSL@HMSN were drastically decreased to 0.2476 cm^3^/g and 235.13 m^2^/g after CSL-loading. After modification with Cs, the V_pore_ and S_BET_ of CSL@HMSNs-CS were further decreased to 0.1205  cm^3^/g and 52.816 m^2^/g. All these changes were results of the obstruction of pore with Cs.

The zeta potentials of the HMSNs were measured to be − 32.8 ± 1.3 mV, suggesting that HMSNs has great dispersity. The zeta potentials of CSL@HMSNs were − 9.5 ± 0.7 mV while the CSL@HMSNs-CS had positively charged with zeta potentials of 19.9 ± 0.7 mV possibly due to the positively charged Cs modification.

**Fig. 3:**
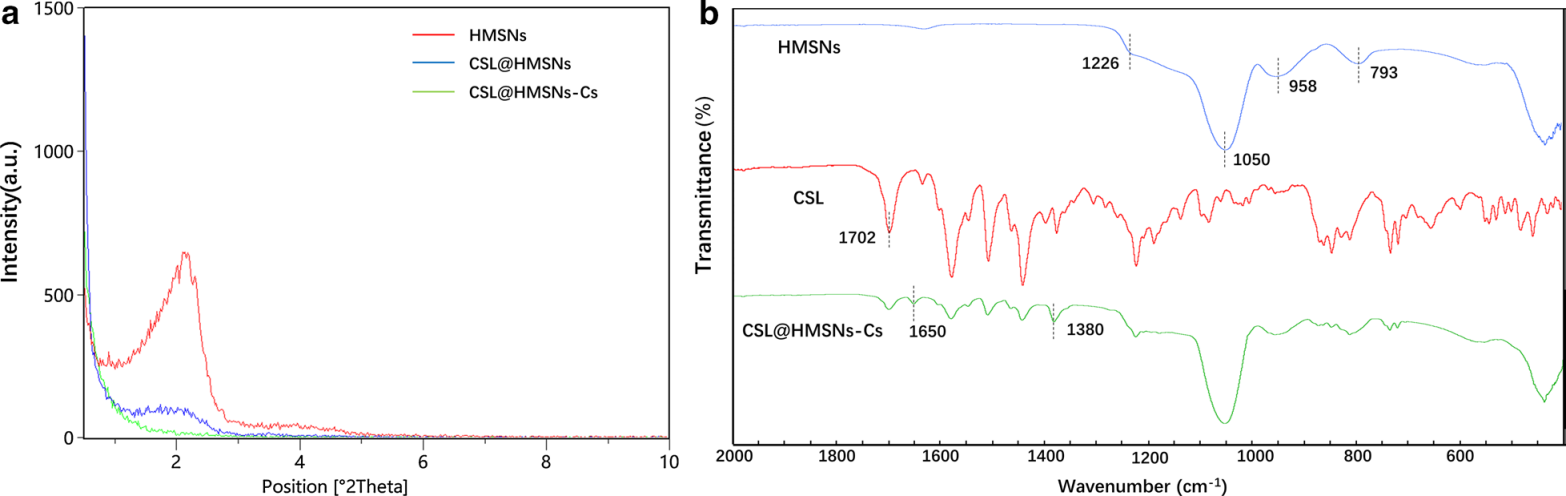
Characterization of HMSNs, CSL@HMSNs and CSL@HMSNs-CS (CHC). Low-angle X-ray diffraction (XRD) patterns (**a**) and Fourier transform infrared (FT-IR) spectra (**b**)

Low-angle X-ray diffraction (XRD) patterns measurement showed that HMSNs exhibits a diffraction peak at 2.1° (2θ) and a weak, broad shoulder peak at 3.9° (Fig. 3a), indicating the existence of wormhole mesostructures [20]. CSL@HMSNs exhibit a same diffraction peak but lower intensity. CSL@HMSNs-CS exhibit no diffraction peak due to the surface modification of Cs.

Fourier transform infrared (FT-IR) spectra (Fig. 3b) showed four characteristic absorption peaks of HMSNs at the peaks at 1226 cm^−1^ and 1050 cm^−1^ corresponding to the asymmetric stretching vibrations of Si–O–Si; 793 cm^−1^ and 958 cm^−1^ corresponding to the symmetric stretching vibration of Si–O–Si and the stretching vibration of Si–OH, respectively. As a result of loading CSL in the cavity, a new peak was detected at 1702 cm^−1^ corresponding to the vibrations of C=O. The characteristic absorption peak of Cs at 1650 and 1380 cm^−1^ indicated the successful coating of the CS layer [18].

All of the above results indicate that the CSL was successfully capped in the cavity with Cs. Functional modification could provide protection for the loaded drug until the nanoparticles were delivered to the target.

To evaluate the time stability, CHC was tested with Fourier transform infrared (FT-IR) spectra 3 months after the nanocomplex was synthesized. The result showed that the main functional group, including Si–O–Si, Si–OH, and characteristic absorption peak of Cs and CSL were not changed during the time (Additional file 1: Figure S2).

The temperature stability of CHC was measured by Thermogravimetric Analysis (TGA) and differential scanning calorimetry (DSC) (Additional file 1: Figure S3). The TGA curve showed two decompositions stage of CHC. The first decomposition was observed within 50–150 °C, with about 10% weight loss by the evaporation of water. The second decomposition appeared within 200–450 °C with about 25% weight loss by decomposition of organic ingredients including CSL and Cs.

The DSC curve of CHC was obtained at a heating rate of 30 °C/min ranged from room temperature to 800 °C under the air atmosphere. No characteristic melting peak of CSL was found in the curve, indicating the CSL in the cavity was in an amorphous state.

### Evaluation of CSL loading capacity, solubility and pH-responsive release in different buffer solutions (pH = 6.0, 6.8, 7.7)

**Fig. 4:**
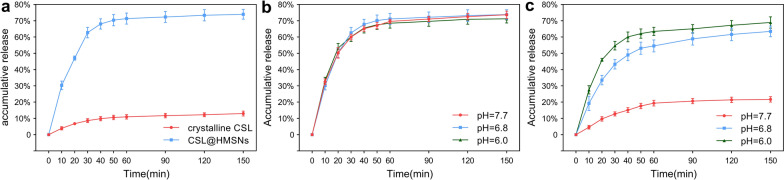
Loading CSL with HMSNs improved the solubility of CSL and capped with Cs gives a pH-responsive property. Solubility and pH-responsive release of CSL, CSL@HMSNs and CSL@HMSNs-CS (CHC). The solubility of crystalline CSL and CSL@HMSNs in pH = 7.4 PBS (**a**). Solubility curve of CSL@HMSNs in different pH PBS (pH = 6.0, 6.8, 7.7) (**b**). Accumulative release curve of CSL@HMSNs-CS in different pH PBS (pH = 6.0, 6.8, 7.7) (**c**)

Hollow spheres could load large amounts of drugs in the cavity. The difference between the amount of CSL initially employed and the content in the supernatant after stirring was defined as the loading content. The loading content of CSL in CSL@HMSNs was determined to be 28.2% although due to inevitable leakage during the process of surface modification, the content decreased to 24.3% in CSL@HMSNs-CS. The pores of HMSNs are much larger than the size of the drug molecules and provide sufficient space for drug free diffusion of the drug into and out of the carrier. The result of CSL solubility (Fig. 4a) showed that crystalline CSL had poor solubility of approximately 12.96% in pH = 7.4 PBS. Loading CSL with HMSNs could largely improve the solubility of CSL to 73.99%.

Approximately 40% or more of new chemical entities are poorly soluble in water, hindering their clinical application [21]. Celastrol is a red acicular crystal with poor water solubility. Disruption of the highly ordered crystalline structure is the rate determining step, which requires high energy [22]. Improvements in drug dissolution can be obtained via the conversion of poorly dissolved crystalline drugs from the crystalline phase to the amorphous form when drugs are loaded into the HMSNs [23].

Targeted controlled release property has been considered a significant characteristic of an expected carrier. The pH-responsive release pattern was conducted in three different pH buffer solutions (pH = 6.0, 6.8, 7.7) simulating physiological (pH = 7.7) and different degrees of osteoarthritis conditions (pH = 6.0, 6.8). In noncoated CSL@HMSNs, a rapid burst release was observed from 0 to 30 min, and then the curve reaches a plateau state (Fig. 4a). Approximately 70% of CSL was released within 60 min from CSL@HMSNs with no significant difference at pH 6.0, 6.8 or 7.7 (71.2%, 73.7%, 73.9%). In contrast, in CSL@HMSNs-CS burst release was not found after stirring for 150 min at pH 7.7 and only 21.7% of CSL was released (Fig. 4c), indicating the great stability of nanoparticles in physiological environments. The cumulative release amount of CSL from CSL@HMSNs-CS was higher at pH 6.0 (68.9%) than at pH 6.8 (63.5%) and pH 7.7 and the cumulative release curve in acidic environment was similar to that of uncoated nanoparticles.

Degradation of the extracellular matrix (ECM) is a key step in the pathological process of OA. During the process, the pH of the synovial fluid of osteoarthritic joints is acidic because of the inflammatory reaction. The insufficient oxygen supply and increased metabolic activity switch toward metabolism towards glycolysis, leading to the accumulation of lactate [24]. The presence of NH_2_ groups on chains of CS provides the possibility for functional modification to HMSNs. Restricted to alkaline environments, the Cs forms a gel-like structure that remains insoluble, forming a protection layer to reduce drug leakage. Osteoarthritis can provide acidic environments to protonate free NH_2_ groups and improve the water solubility of Cs. The CS layer swells, leading to the exposure of pore entrances and the release of drugs was triggered [18]. Medical Cs are intra-articular injected for clinical application, given their protective action on osteoarthritis by preventing type II collagen degradation [25]. In the knee joint, Cs could be degraded by lysozymes, which physiologically exist in human cartilage and the main degradation product is glucosamine, preventing type II collagen from degradation clinically [26, 27]. The pH-responsive property and high biodegradability of Cs make it an ideal gatekeeper for knee intra-articular injection. There were no pathological changes in the present study compared with HMSNs with HMSNs-Cs, which may be because the concentration of Cs was not high enough for clinical treatment.

### In vitro cell viability and cytotoxicity assay

**Fig. 5:**
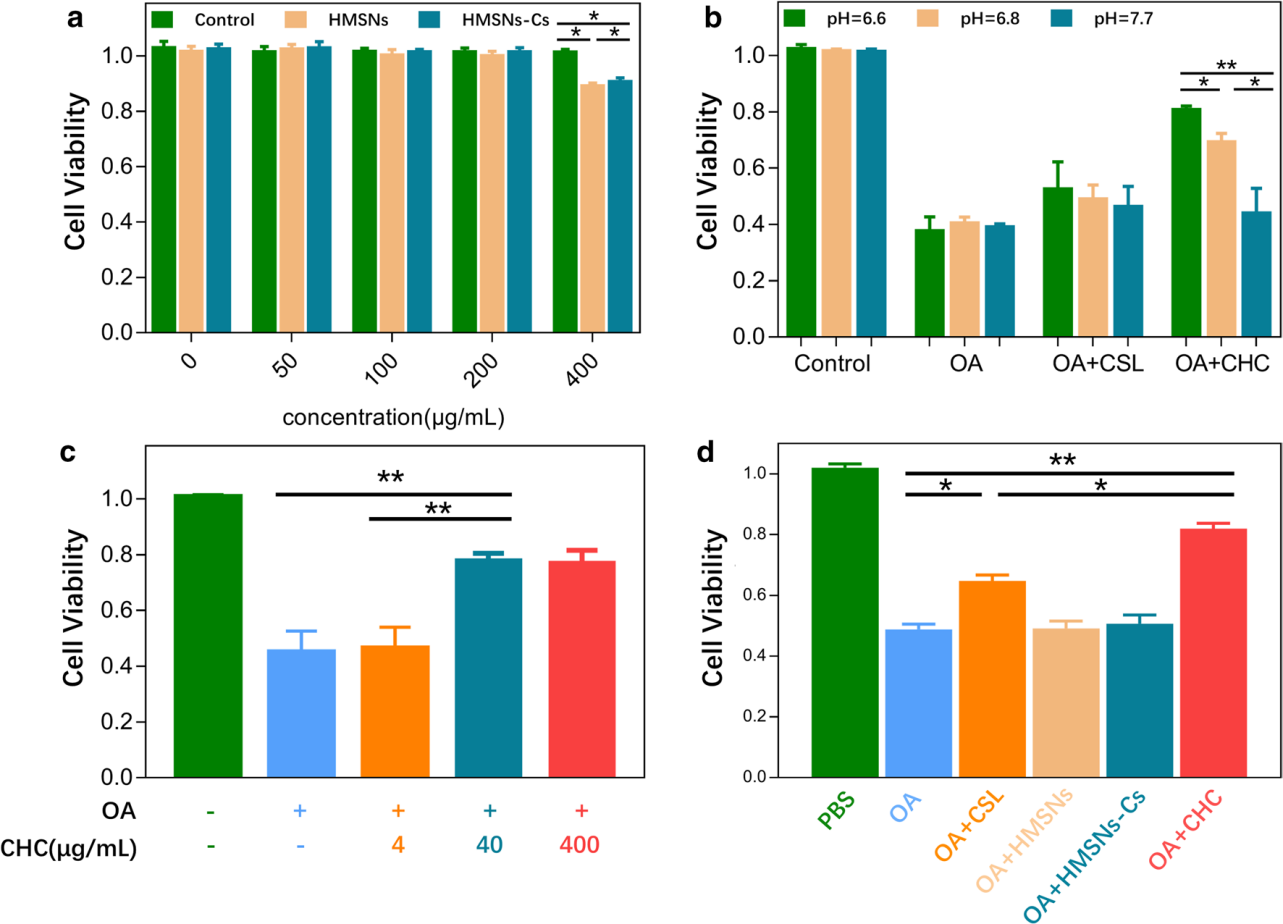
**a** in vitro cytotoxicity of different concentrations of HMSNs and HMSNs-Cs. **b** cell viability of CSL@HMSNs-CS (CHC) applied in IL-1β stimulated chondrocytes in the different pH culture medium. **c** concentration-dependent therapeutic effects of different concentrations of CSL@HMSNs-CS. **d** cell viability of CSL, HMSNs, HMSNs-Cs, CSL@HMSNs-CS (CHC) applied in IL-1β stimulated chondrocytes (*p < 0.05; **p < 0.01)

The nanoparticle cell viability assay showed no significant cytotoxicity at relatively low concentration (50, 100, 200 μg/mL) of HMSNs and HMSN-Cs after incubation for 3 h and 24 h. There was no significance among each groups (Additional file 1: Figure S4). After 24 h incubation, when the concentration of HMSNs and HMSN-Cs increased to 400 μg/mL, obvious cell cytotoxicity was observed (Fig. 5a). These results suggested that HMSNs and HMSNs-Cs showed great biocompatibility. In Fig. 5b, the administration of CSL@HMSNs-Cs increases the cell viability of chondrocytes after stimulation by IL-1β in pH 6.0 culture medium compared with pH 6.8 and pH 7.7, indicating the pH-responsive treatment of CSL@HMSNs-CS. In Fig. 5c, the data of concentration-dependent therapeutic effects showed that at low concentration (4 μg/mL, approximately 1 μg/mL CSL after calculated by the loading capacity of CSL@HMSNs-CS) of CSL@HMSNs-CS, there is no difference compared with OA groups. At the concentration of 40 μg/mL, the cell viability was increased but no significant difference compared with 400 μg/mL group.

A cell viability assay was applied to examine the therapeutic effect of CSL and CSL@HMSNs-CS (Fig. 5d). After stimulation with IL-1β to simulate the OA condition, compared with the control group without nanoparticle intervention, the cell viability was decreased. Treatment with simple CSL slightly increased the cell viability, whereas notable growth was observed after treatment with CSL@HMSNs-CS compared with OA condition. The data of the HMSNs and HMSNs-Cs group showed no significant difference with the OA condition.

All these results demonstrated the pH-responsive property of CSL@HMSNs-CS and that cell viability could be reversed by treatment with CSL and CSL@HMSNs-CS. The data of the in vitro cell viability assay demonstrate an improvement in CSL@HMSNs-CS compared with simple CSL, indicating that the HMSN loading and the Cs coating provide an extraordinary bioavailability. In the laboratory, to prepare drugs with poor solubility, solvent dimethyl sulfoxide (DMSO) was often chosen as a virtual ‘universal solvent’. But the cell permeabilizing effects of DMSO may influence the results. HMSNs are excellent candidates for loading traditional medicine facing the issue of their poor solubility. The ability to improve bioavailability has been demonstrated in many drugs with poor solubilities such as resveratrol and albendazole [28, 29]. The high volume of pores provides a large contact area to the vehicle solution, leading to better drug loading and release. The high loading capacity of HMSNs could reduce potential cytotoxicity of CSL owing to the rapid release of the drug.

### Enzyme-linked immunosorbent assay (ELISA)

**Fig. 6:**
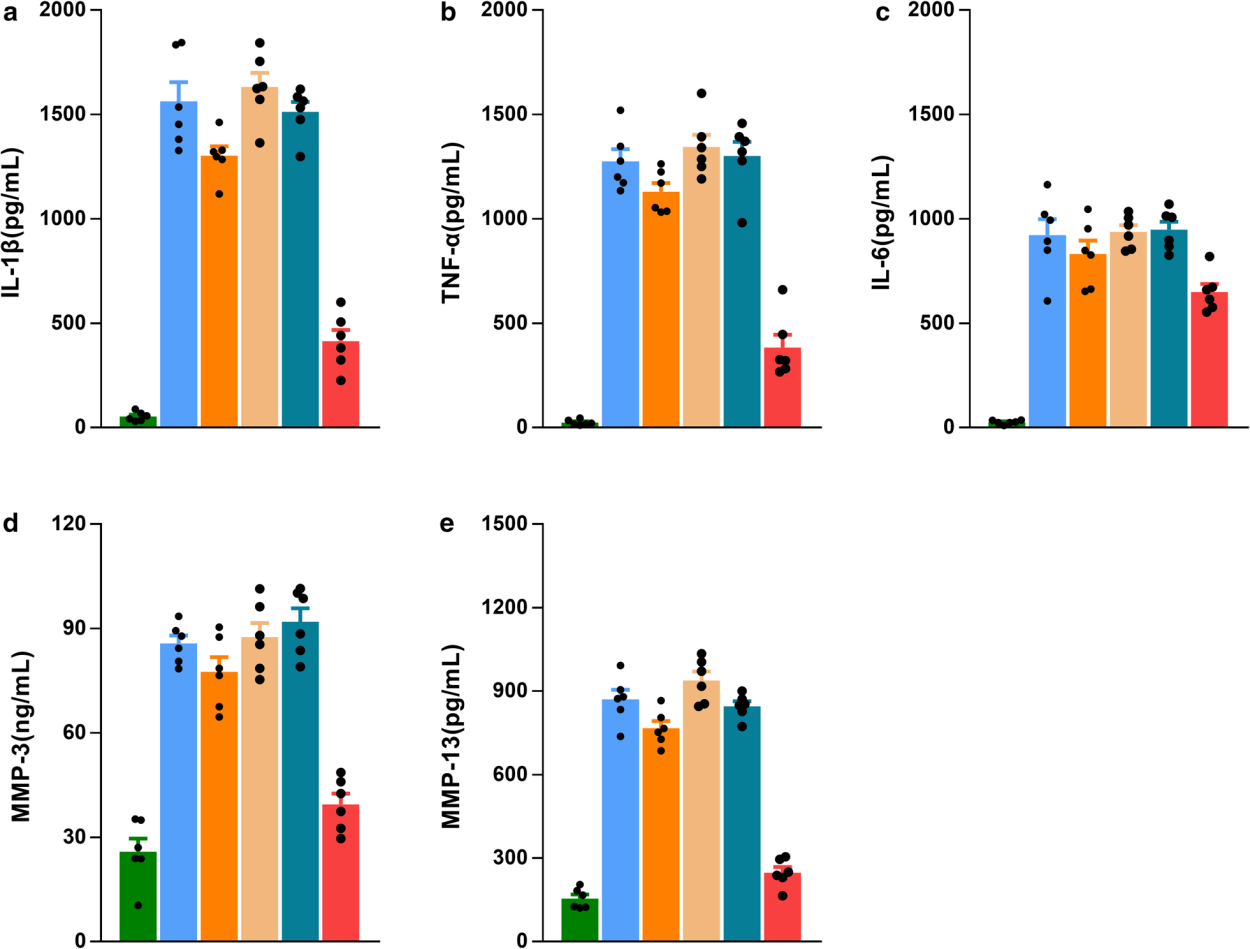
ELISA assay of expression levels of IL-1β (**a**), TNF-α (**b**), IL-6 (**c**), MMP-3, MMP-13 in chondrocyte supernatant stimulated by IL-1β for 1 h followed by incubation with complete medium with CSL, HMSNs, HMSNs-Cs and CSL@HMSNs-CS (CHC) at equivalent CSL concentrations (10 μg/mL) for 24 h. (*p < 0.05; **p < 0.01)

The expression levels of inflammatory factors and MMP-3, MMP-13 in chondrocyte supernatant were evaluated via ELISA (Fig. 6). The expression levels of IL-1β, IL-6, TNF-α, MMP-3, and MMP-13 dramatically increased in the OA, HMSNs, HMSNs-Cs groups, with no significant differences among the groups. In the simple CSL group, the expression level was slightly decreased, and a further decrease was observed in the CSL@HMSNs-CS group, revealing the good anti-inflammatory effect of nanoparticle medicine.

The increase in inflammatory factors probably due to the activation of pro-inflammatory enzymes involved in the processing of inflammatory factor precursor synthesis. The present study showed that Inflammatory factors, which are part of a vicious cycle in OA progression, could be inhibited by CSL@HMSNs-CS. Inhibition of IL-1β and TNF-α can block the OA occurrence and the development of the early and late course of OA, as well as the infiltration of immune cells and the destruction of cartilage structural integrity.

Matrix metalloproteinases (MMPs) are a broad family of secreted or transmembrane zinc-dependent endoproteinases that play a significant role in the degradation of ECM [30]. Among them, MMP-3 and MMP-13 are powerful collagenolytic enzymes that show proteolytic activity on type II collagen, which is the most abundant protein component of cartilage and maintain morphology and function of cartilage [31]. Stimulation by inflammatory reactions could induce the secretion of these enzymes from cartilage and synovial cells in the early stages of OA. Overexpression of proteolytic enzymes triggered an increase in the breakdown products from the ECM, leading to the phagocytosis by the synovial cells. Such positive-feedback regulation may amplify the development of OA [32]. Using an integrated systems pharmacology method, it was predicted that the MMPs family is the direct target of CSL in rheumatoid arthritis, partially involved in the therapeutic effects in rheumatoid arthritis [33]. The results of the present study indicated that CSL@HMSNs-Cs provide an ideal treatment by downregulating the expression of MMP-3 and MMP-13, which could be responsible for the anti-inflammatory effect previously mentioned.

### Evaluation of pain behavior

**Fig. 7:**
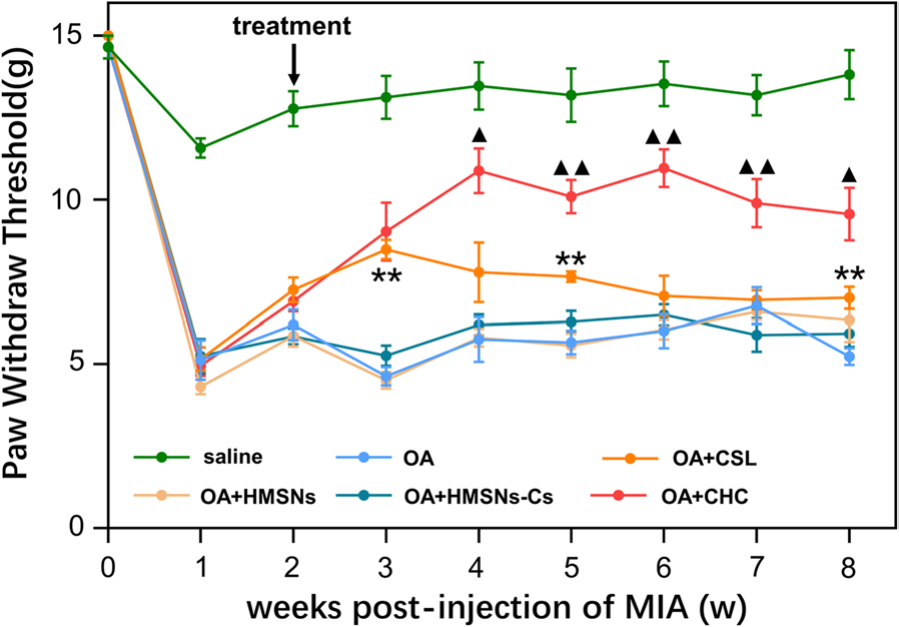
Paw withdrawal threshold (PWT) of MIA-induced OA rats treated with CSL, HMSNs, HMSNs-Cs and CSL@HMSNs-CS (CHC). CSL@HMSNs-CS significantly upper the PWT of OA rats. (**, comparison with saline group, *p *< 0.01; ▲, comparison between the groups treated with simple CSL and CSL@HMSNs-CS (CHC), *p *< 0.05; ▲▲, comparison between the groups treated with simple CSL and CSL@HMSNs-CS (CHC), *p *< 0.01)

Von Frey filaments are the gold standard way of evaluating sensory thresholds. Mechanical sensitivity is expressed by the paw withdrawal threshold (PWT) upon pricking the hind limb with von Frey filaments. The up-down method was used to estimate and modify thresholds [34, 35]. According to the results of the pain behavior test (Fig. 7), the PWT decreased in the 1st week after MIA injection. In the 2nd week, drug intervention was conducted via intra-articular injection. After that, an increase in PWT was observed in both the CSL and CSL@HMSNs-CS groups. A significant difference between the CSL and CSL@HMSNs-CS groups was demonstrated after the 4th week, showing the effect of inhibiting central sensitization.

Pain is the major symptom of OA and is involved in both peripheral nociceptive stimuli and central sensitization. The acidic environment in OA could activate the osteoclasts, leading to the attenuation of the subchondral plate. The explosion of subchondral nerves because of the destruction of osteochondral integrity induces a continuous peripheral stimulation [36]. In addition, sensory neurons are sensitive to H^+^, so nociceptive sensory neurons could be directly excited by H^+^-gated currents in the acidosis extracellular environment [37]. The acute inflammatory response caused by the injection of MIA would be resolved by week 1 but could give rise to the sensitization of peripheral receptors. Increased stimulation input from peripheral nociceptors enhances the excitability of dorsal horn neurons at the dorsal horn, the so-called central sensitization, leading to the mechanical hypersensitivity [38]. CSL could relieve knee OA pain by decreasing cytokines expression, inhibiting inflammatory infiltration and reducing peripheral stimulation, and interrupting the process of pain formation.

### MRI and safranin O fast green staining of the knee joint

**Fig. 8:**
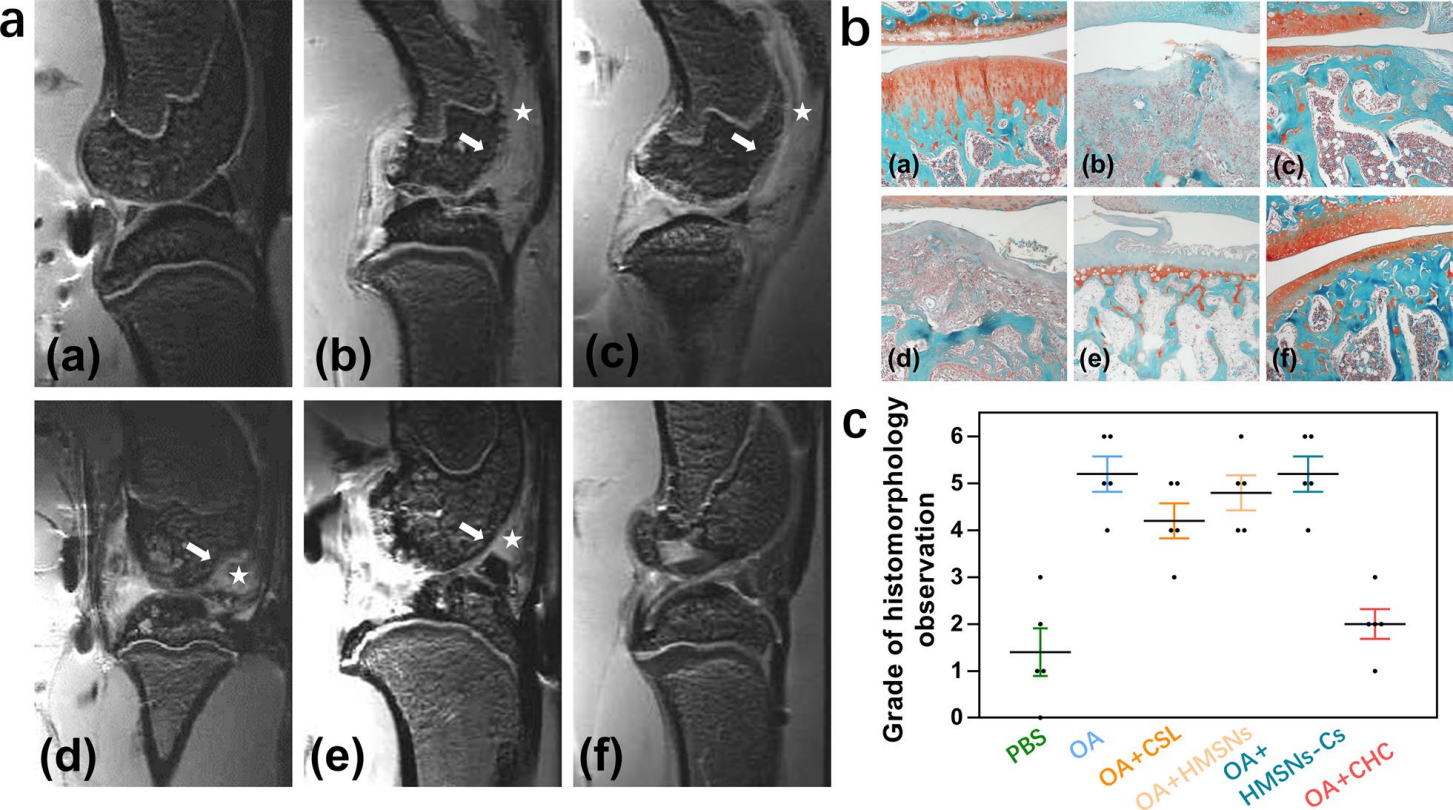
**A** MRI images of the knee joints. (a) saline; (b) OA; (c) CSL; (d)HMSNs; (e) HMSNs-Cs; (f) CSL@HMSNs-CS (CHC). Articular surface erosion was marked by arrow and prominent joint effusion was marked by star. **B** Safranin O Fast Green staining. (a) saline; (b) OA; (c) CSL; (d)HMSNs; (e) HMSNs-Cs; (f) CSL@HMSNs-CS (CHC). The proteoglycan in cartilage martrix stains red and the collagenous fiber in bone stains blue or green. **C** Cartilage damage was measured on Safranin-O Fast Green staining histological slides by a modified OARSI score system (n = 5)

MRI images of the knee joints (Fig. 8a) revealed that articular surface erosion (marked by arrow) and prominent joint effusion (marked by star) were present in the OA group. The images showed no obvious effect on osteoarthritis in the simple CSL group, whereas a great improvement in articular surface erosion and joint effusion was observed in the CSL@HMSNs-CS group, indicating extraordinary therapeutic efficacy. The safranin O fast green staining result showed the same pathological changes as observed by MRI (Fig. 8b). Comparing with the saline group, the OA group showed obvious vast cartilage loss, disorganized chondrocytes and structural destruction. Injection of simple CSL could improve the changes, but the cartilage layer is thinner and shows fissures. A dramatic improvement in pathological changes, such as smooth cartilage surface, undulating tide line and cartilage thickness was observed in the CSL@HMSNs-CS group.

Intra-articular injection of monosodium iodoacetate is a commonly used method to build an OA animal model to imitate human OA cartilage and bone pathology [39]. At 2 weeks post-injection, a high dosage of MIA (2 mg) could simulate the pathological characteristics of subchondral bone and subchondral trabecular bone, such as erosion of subchondral trabecular bone, destruction of articular cartilage, and exposure of subchondral bone, similar to end-stage human OA joints [40]. Subchondral bone stiffening and the destruction of articular cartilage play a critical role in the progression of OA. The dynamic balance between osteoclasts and osteoblasts leads to disorganized cartilage and bone structure. The image of the OA group showed severe loss of joint cartilage and remodeling of subchondral bone. CSL, forming an insoluble precipitate, can lower joint lubrication and induce the secretion of inflammatory factors in cartilage. According to the MRI results, a profoundly reduced knee swelling and improvement in synovial inflammation and cartilage integrity were demonstrated in the CSL@HMSNs-CS group, suggesting a protective effect on the structure of cartilage and subchondral bone.

### Protein expression of NF-κB signalling pathway

**Fig. 9:**
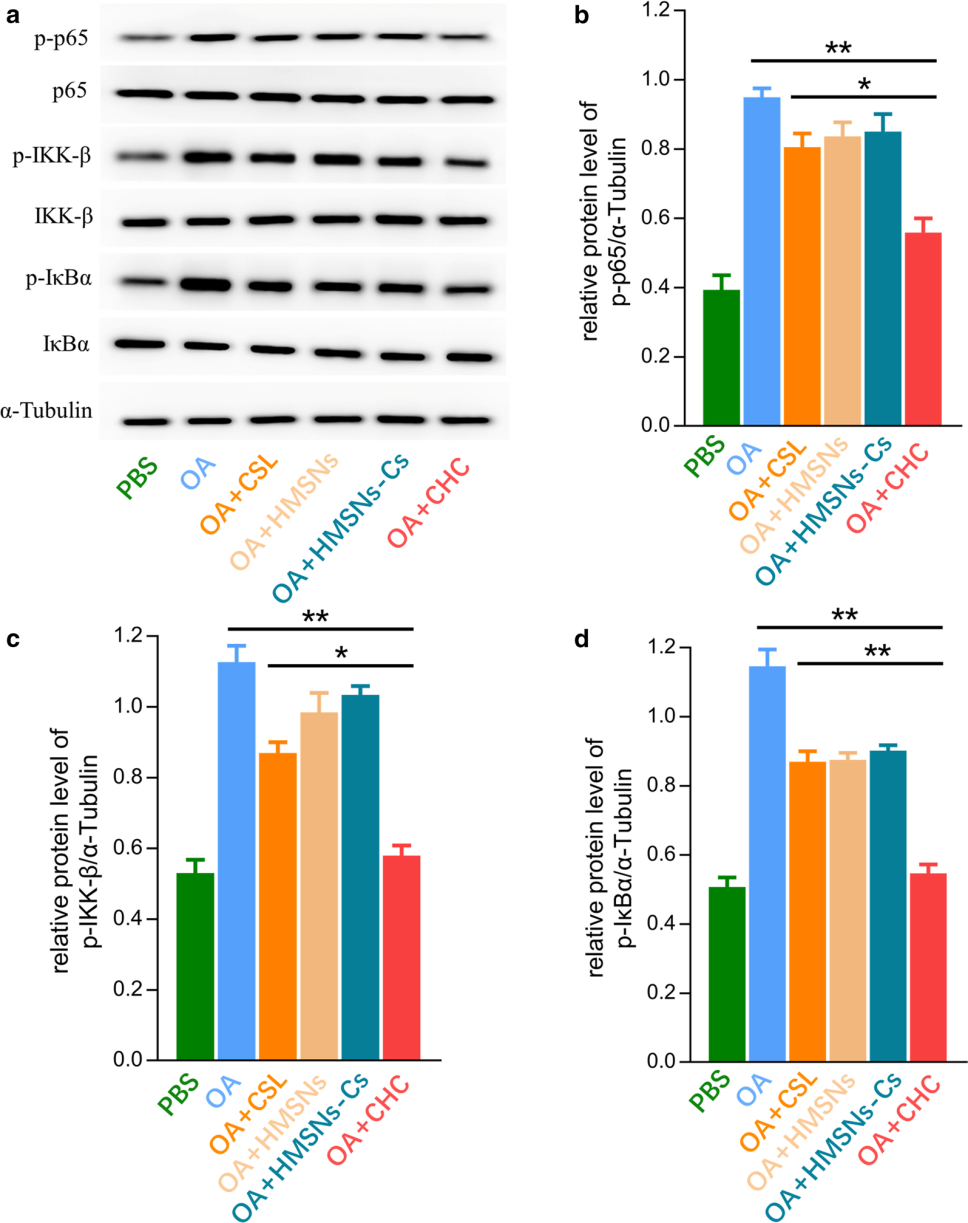
Protein expression levels of p-p65, p-IKKβ and p-IκBα of chondrocytes stimulated by IL-1β after drug intervention with CSL, HMSNs, HMSNs-Cs and CSL@HMSNs-Cs (CHC). **a** the protein expression levels of p65, p-p65, IKKβ, p-IKKβ, IκBα and p-IκBα were measured by western blot. **b**–**d** protein expression levels of p-p65, p-IKKβ and p-IκBα (*, p < 0.05; **, p < 0.01)

The protein expression levels of phosphorylated p65, phosphorylated IKKβ and phosphorylated IκBα were demonstrated in Fig. 9. Compared with the control group, the expression levels of phosphorylated p-65, phosphorylated IKKβ and phosphorylated IκBα were dramatically up-regulated in OA, HMSNs, HMSNs-Cs group. Simple CSL could downregulate the expression level but is of limited effectiveness. In CSL@HMSNs-CS group, the protein expression level was significantly downregulated suggesting the notable effect in inhibiting the expression level of the NF-κB signalling pathway in chondrocytes.

Celastrol has various of anti-inflammatory cellular targets and can significantly reduce the upregulated expression of MMPs family and protect chondrocytes against the IL-1β-induced inflammatory response and apoptosis [41]. NF-κB signalling pathway is a typical signalling pathway involved in the development of OA pathobiology. Cartilage degradation can be induced by NF-κB transcription factors to enhance the secretion of various degradative enzymes such as MMP and A disintegrin and metalloproteinase with thrombospondin motifs (ADAMTS) which play a critical role in the degradation of ECM structural protein [42, 43]. CSL attenuates NF-κB translocation to the nuclear, and pretreatment with CSL reduces the matrix degradation induced by IL-1β [44]. IκBα phosphorylation by the IKK complex can be inhibited by celastrol in different cell types, which is a key step in NF-κB activation, consistent with the present study [45].

## Conclusion

In summary, this study provided new insights into the therapeutic effects of intra-articular injection of celastrol-loaded, chitosan packaged hollow mesoporous silica nanoparticles against osteoarthritis by reducing cartilage damage and relieving inflammation in vitro and in vivo. HMSNs could improve the solubility of CSL and surface conjugation of Cs gives the property of pH-response, enhancing the bioavailability of CSL. The nanoparticles have proven effective, pH-responsive and safe against osteoarthritis by animal behavior tests, histopathological observation and diagnostic imaging tests. In the future, hollow mesoporous silica nanoparticles should be considered potential candidate nanocarriers for the intra-articular injection of natural medicines, as they promote a reliable pH-responsibility and improvement insolubility.

## Methods

### Chemicals and regents

All chemicals and reagents were obtained commercially and were analytical grade. Hexadecyl trimethyl ammonium bromide (CTAB), tetraethyl orthosilicate (TEOS), 3-glycidoxypropyltrimethoxysilane (GPTMS), aqueous ammonia, sodium carbonate, ethanol, acetic acid and methyl alcohol were obtained from Sinopharm Chemical Reagent Co, China.

0.2% trypsin was obtained from Hyclone, USA. Dulbecco’s modified Eagle’s medium (DMEM) and 1% penicillin/streptomycin were obtained from Thermo Fisher Scientific, USA. Monosodium iodoacetate (MIA) was obtained from Aladdin Industrial Co, China. 10% fetal bovine serum (FBS) and 5% bovine serum albumin (BSA) were obtained from Jackson Immuno Research Inc. USA. Phosphorylated p65, p65, phosphorylated IKKβ, IKKβ, phosphorylated IκBα, IκBα primary antibodies were obtained from Cell Signaling Technology, USA. Collagenase, Celastrol (CSL) and interleukin 1β (IL-1β) were obtained from Sigma-Aldrich, USA. Ethylenediaminetetraacetic acid (EDTA), 10% paraformaldehyde, horseradish peroxidase (HRP)-conjugated secondary antibody, Cell Counting Kit-8 (CCK-8), RIPA lysis buffer, BCA Protein Assay Kit and enhanced chemiluminescence (ECL) detection kit were obtained from Beyotime Institute of Biotechnology, China.

### Synthesis of HMSNs

The “structural difference-based selective etching” method was employed to synthesize HMSNs with some modifications. First, solid SiO_2_ nanoparticles (sSiO_2_) were synthesized via the Stöber method. Ammonia aqueous (3.14 mL) was dissolved into an ethanol/water (74 mL/10 mL) mixture. TEOS was added to the mixture, which was stirred at 30 °C for 2 h. sSiO_2_ nanoparticles were obtained after centrifugation and then washed with ethanol and distilled water each for three times. Next, the core/shell nanoparticles were further synthesized. The sSiO_2_ nanoparticles (500 mg) were dispersed in distilled water (100 mL) via ultrasonication for 20 min. A solution of aqueous CTAB/water/ethanol/ammonia aqueous (0.75 mg/150 mL/150 mL/2.75 mL) was mixed with sSiO_2_ suspension. CTAB was absorbed to the surface of sSiO_2_ after ultrasonic for 2 h, and then TEOS (1.5 mL) was added and continue to ultrasonicate. The product (SiO_2_@CTAB-SiO_2_) was obtained after centrifugation and sequentially rinsed with ethanol and water three times. Finally, we formed a hollow structure in the silica nanoparticles via a selective etching method. The product obtained by the above procedure was dissolved in sodium carbonate (Na_2_CO_3_, 0.6 M, 50 mL) and stirred at 80 °C for 6 h. After centrifugation and rinsing, the crude product was then suspended in a mixture of methanol/hydrochloric acid (1 g/180 mL/1 mL) and stirred at 80 °C for 6 h, repeated three times. The final product (HMSN) was obtained after centrifugation and rinsed with distilled water for three times.

### Synthesis of CSL@HMSNs-CS

CSL@HMSNs-Cs were prepared according to the previous reports [46, 47] with some modifications. First, GPTMS was employed to epoxy-functionalize the HMSNs. One hundred milligrams of HMSNs were dispersed in 5 mL toluene and the pH of the solution was adjusted to 3.5–4.5. Then, 100 mg of GPTMS was added to the mixture and refluxed for 6 h in a nitrogen atmosphere. Then, the crude product (HMSN-GPTMS) was obtained after centrifugation, rinsed with toluene water three times and dried at 100 °C overnight. Next, CSL was loaded into the HMSN-GPTMS by simple diffusion. Dried HMSN-GPTMS was dispersed in 10 mL CSL/ethanol solution (15 mg/mL) and stirred while shielded from direct sunlight at 25 °C for 24 h. CSL@HMSN-GPTMS were collected by centrifugation, water washing, and freeze-drying. Finally, GPTMS was reacted onto chitosan chains via the acid-catalyzed amino-oxirane addition reaction. Cs was dispersed into 10 mL of acetic acid (10%, v/v) and stirred at 25 °C for 48 h. After adjusting the pH value of Cs-acetic acid collosol to 5.0, freeze-dried CSL@HMSN-GPTMS was added and stirred for 12 h. Then the pH value of the mixture solution was adjusted to 7.4 with Sodium hydroxide (NaOH, 1 M) and remain stirred for 2 h. CSL@HMSNs-CS were collected by centrifugation, washed three times with phosphate-buffered saline (PBS) at pH 7.4 for followed by freeze-drying.

### Characterization of CSL@HMSNs-CS

The morphology of the samples was observed on a JEM-2011 (JEOL Ltd., Japan) electron microscope at a working voltage of 200 kV. The Fourier transform infrared spectrophotometric (FT-IR) spectra were determined on a Nicolet 6700 FT-IR spectrometric analyzer using KBr discs in the region of 2000–400 cm^−1^. The low-angle X-ray diffraction (XRD) patterns were obtained by a D/max2550VB3 + /PC (Rigaku Co., Japan) X-ray diffractometer with Cu Kα radiation (λ = 0.15418 nm). The operation voltage and current were maintained at 40 kV and 40 mA. The XRD patterns were recorded in the range of 0.5°–10° and the scan rate was 0.01°/s. The nanoparticle sizes and zeta potentials were determined by a Malvern Zetasizer Nano-ZS90. Nitrogen adsorption–desorption experiments were carried out on a Micromeritics Tristar 3000 analyzer at 77 K to measure the porous structure. The surface area, pore volume and pore size were obtained using the Brunauer–Emmett–Teller (BET) and Barrett–Joyner–Halenda (BJH) analyses, respectively.

### Evaluation of CSL loading capacity, solubility and pH-responsive release in different buffer solutions (pH = 6.0, 6.8, 7.7)

The loading capacity of CSL incorporated in CSL@HMSNs-CS was measured by an ultraviolet spectrophotometer before and after the encapsulation of CSL in EtOH. The calibration curve was obtained from the average reading of different CSL concentrations in the EtOH solution (λmax = 424 nm, r = 0.9989). HMSNs were dissolved in CSL/EtOH solution followed by stirring for 24 h. The supernatant liquor was collected after centrifugation of the obtained CSL@HMSNs. After filtration through a 0.45 mm membrane filter, the filtrate was determined with an ultraviolet spectrophotometer at 424 nm to measure the amount of CSL loaded in HMSNs. The same methods were used to measure the amount of CSL leaked during the Cs-capping process. The CSL loading capacity in CSL@HMSNs was calculated according to the following equation: CSL loading capacity (%, w/w) = mass of CSL loaded in CSL@HMSNs/mass of CSL@HMSNs × 100%. The same equation was applied to calculate the CSL loading capacity in CSL@HMSNs-CS.

To evaluate the solubility, crystalline CSL and CSL@HMSNs (equivalent CSL amount,10 mg) were added to 50 mL pH = 7.4 PBS. 1 mL supernatant was collected at different time points and the concentration of CSL was determined by ultraviolet spectrophotometer (λmax = 424 nm).

To determine CSL-responsive release in different PBS solutions (pH = 6.0, 6.8, 7.7), the accurately weighted CSL@HMSNs and CSL@HMSNs-CS were resuspended in pH 6.0 PBS, pH 6.8 PBS and pH 7.7 PBS at room temperature. One milliliter of supernatant was collected at predetermined time points, and the amount of CSL released was determined by an ultraviolet spectrophotometer (λmax = 424 nm).

### Cell culture and cellular osteoarthritis model

Cartilage tissues of 14-day-old rats were harvested, cut into pieces and digested with trypsin (0.2%, Hyclone, USA) and collagenase (Sigma-Aldrich, USA). Isolated chondrocytes were cultured in Dulbecco’s modified Eagle’s medium (DMEM, Thermo Fisher Scientific) supplemented with 10% fetal bovine serum (FBS), 1% penicillin/streptomycin (Thermo Fisher Scientific, USA). Cell culture experiments were performed under a stable atmosphere at 37 °C, 5% CO_2_ and 100% humidity. The culture medium was changed every 5 days. Chondrocytes at the P3 stage were used for subsequent assays. To produce a cellular osteoarthritis model, cells were plated in 6-well plates and cultured for 24 h and stimulated with interleukin 1β (IL-1β, 10 ng/mL, Sigma-Aldrich, USA) for 1 h to simulate osteoarthritis.

### In vitro viability and cytotoxicity assay

Cell cytotoxic effects were measured with a Cell Counting Kit-8 (CCK-8, Beyotime Institute of Biotechnology, China). Cells were incubated in complete medium containing different amounts of HMSNs and HMSNs-Cs (from 0–400 μg/mL) for 24 h. Then the medium was removed and the cells were washed with precooling PBS for three times. The CCK-8 solution (10 μL/well) was added to the culture medium and incubated for another 1 h.

To evaluate the nanoparticle treatment, cells were stimulated by IL-1β for 1 h followed by incubation with complete medium with CSL, HMSNs, HMSNs-Cs and CSL@HMSNs-CS at equivalent CSL concentrations (10 μg/mL) for 24 h. Then the medium was removed, and the cells were washed with precooled PBS three times. The CCK-8 solution (10 μL/well) was added to the culture medium and incubated for another 1 h. The absorbance value was recorded in a spectrophotometer at 450 nm. Each intervention was tested in triplicate.

To evaluate the concentration-dependent therapeutic effects of CSL@HMSNs-Cs, chondrocyte was stimulated by IL-1β for 1 h followed by incubation with 4, 40, 400 μg/mL CSL@HMSNs-Cs dissolved in complete medium for 24 h. Then CCL-8 assay was applied as mentioned above. The absorbance value was recorded in a spectrophotometer at 450 nm. Each intervention was tested in triplicate.

The same method was applied to evaluate the therapeutic effect of CSL, HMSNs, HMSNs-Cs and CSL@HMSNs-CS in different pH culture medium which is pre-adjusted by 1 M HCl and 1 M NaOH with a pH detector followed by cell strainers (pH = 6.0, 6.8, 7.7). After stimulated by IL-1β for 1 h, the medium was removed and the cells were washed with precooled PBS. CSL, HMSNs, HMSNs-Cs and CSL@HMSNs-CS at equivalent CSL concentrations (10 μg/mL) was dissolved in different pH culture medium and incubated for 24 h. The cell viability of chondrocytes was measured with the previous mentioned method.

### Enzyme-linked immunosorbent assay (ELISA) of inflammatory cytokines and MMP (Matrix metalloproteinases)-3, MMP-13

The secretion of IL-1β, TNF-α, Il-6 and MMP-3, MMP-13 in chondrocyte supernatant were measured with specific ELISA kits (R&D Systems, USA) according to the manufacturer’s instructions.

### Animals

Six-week-old male Sprague–Dawley rats (220–240 g) were obtained from Shanghai SLAC Laboratory Animal Co., Ltd. (China). All animal procedures were performed under the protocols approved by the Ethics Committee of Xinhua Hospital Affiliated with Shanghai Jiao Tong University School of Medicine. Animal experiments were performed in accordance with the guidelines of the National Institute of Health Guide for the Care and Use of Laboratory Animals.

### Osteoarthritis model and pharmacological interventions

Osteoarthritis was induced in the left knee by intra-articular injection of monosodium iodoacetate (MIA, 2 mg/50 µL) in sterile 0.9% normal saline, pH 7.4, after anaesthetization. Nonosteoarthritic rats were used as controls. Fifty microliters of intra-articular pharmacological interventions (saline, CSL, HMSNs, HMSNs-CS, CSL@HMSNs-CS) were administered 2 weeks after OA induction with equivalent CSL concentrations (10 μg/mL), respectively (n = 6). 6 weeks after the pharmacological interventions, rats were killed by asphyxiation in carbon dioxide and the knee joints, liver and kidney were harvested for analysis. All outcome measurements were carried out by an experimenter blinded to the randomized treatments.

### Evaluation of pain behavior

Assessment of pain behavior was evaluated by measuring the mechanical withdrawal threshold every week before and after OA induction. Briefly, the rats were placed in a Plexiglas box with a screen platform and allowed to acclimate to the environment for 10 min. A series of calibrated von Frey monofilaments of various bending forces were applied in ascending order of bending force to the plantar surface of the hind paw and held for approximately 6–8 s. A positive response was defined as paw withdrawal occurring twice in the 10 applications. When negative responses occurred, a larger force monofilament was applied. The mechanical withdrawal threshold was calculated according to the S.R. Chaplan’s protocol [48].

### Magnetic resonance imaging (MRI) of the knee joints

For magnetic resonance imaging, the rats were anesthetized and Intra-articular injected knees were imaged by a Bruker Biospec 117/16 USR system, transmit-receive quadrature coil with the following RARE-MRI sequence: echo time = 10 ms, repetition time = 2500 ms, echo spacing = 10 ms, slice thickness = 0.5 mm, image size = 384 × 256, field of view = 25.6 × 17.2 mm, number of excitation = 1, flip angle = 90°, bandwidth = 37.879 kHz, and scan time = 10 min 40 s.

### HE and safranin O fast green staining of the knee joints

Rat knee joints were fixed in 10% paraformaldehyde for 24 h and decalcified in 20% ethylenediaminetetraacetic acid (EDTA) solution for 2 months. Then, the specimens were dehydrated, infiltrated with paraffin, and embedded in paraffin wax. Subsequently, the specimens were sectioned into 5.0 μm sections along the sagittal plane and then stained with HE and Safranin O fast green. Histological changes were observed under a light microscope. To evaluate the cartilage damage, the modified OARSI scoring system was used (n = 5) [49].

### Western-blot analysis

Chondrocyte protein was extracted with RIPA lysis buffer (Beyotime Institute of Biotechnology) with protease inhibitors on ice. The extracted protein concentration was measured with a BCA Protein Assay Kit (Beyotime Institute of Biotechnology) using a microplate reader (Thermo Multiskan FC, Thermo Fisher Scientific, USA). Then the protein was loaded on SDS-PAGE-denaturing gels followed by gel electrophoresis. Then the membrane was blocked in 5% bovine serum albumin (BSA, Jackson Immuno Research Inc.) for 1 h at room temperature to block nonspecific binding and incubated with the phosphorylated p65, p65, phosphorylated IKKβ, IKKβ, phosphorylated IκBα, IκBα primary antibodies (Cell Signaling Technology, USA) at a 1:1000 dilution overnight at 4 °C. Subsequently, after washing in Tris-buffered saline with Tween (TBST), the membrane was incubated in horseradish peroxidase (HRP)-conjugated secondary antibody diluted 1:1000 in 5% (w/v) BSA solution for 1 h at room temperature. Detection was performed using an enhanced chemiluminescence (ECL) detection kit (Beyotime Institute of Biotechnology) according to the manufacturer’s protocol. The bands were analyzed using an imaging system (ChemiScope 6000 Exp, Clinx Science Instruments Co. China). Quantification of the signal intensity for each band was measured with the ImageJ software.

### Statistical analysis

All data were analyzed using Prism software (v 7.0, GraphPad Prism Inc., USA). Pain behavior differences between groups were analyzed using Kruskal–Wallis test. The rest of the statistical comparisons were conducted by analysis of variance (ANOVA) followed by Bonferroni post hoc test with SPSS 25. All data are presented as the mean ± SEM. *p* values of < 0.05 were considered statistically significant.

## Supplementary information

**Additional file 1: S1.** Standard curves of CSL/EtOU solution. **S2.** Time stability of CHC, tested with Fourier transform infrared (FT-IR) spectra 3 months after the nanocomplex was synthesized. **S3.** Thermogravimetric Analysis (TGA, black curve) and differential scanning calorimetry (DSC, blue curve). **S4.** Cytotoxicity assay of different concentration (0, 50, 100, 200, 400 μg/mL) after 3 h. There was no significance among each groups. (n = 3, p > 0.05). **S5.** Paw withdrawal threshold (PWT) of rats treated with saline, 50 mg HMSNs, 100 mg HMSNs, total volume 50 μL. **S6.** Haematoxylin and eosin (H&E) stained images of hepatic tissue 8 weeks after administration of saline, 50 mg HMSNs, 100 mg HMSNs. **S7.** Haematoxylin and eosin (H&E) stained images of nephridial tissue 8 weeks after administration of saline, 50 mg HMSNs, 100 mg HMSNs. **S8.** Haematoxylin and eosin (H&E) stained images of knee joint 8 weeks after administration of saline, 50 mg HMSNs, 100 mg HMSNs.

## Data Availability

All data generated or analyzed during this study are included in this published article and its additional file.

## Abbreviations

ADAMTS: A disintegrin and metalloproteinase with thrombospondin motifs
CCK-8: Cell counting kit-8
CHC: CSL@HMSNs-Cs
Cs: Chitosan
CSL: Celastrol
CTAB: Hexadecyl trimethyl ammonium bromide
ECM: Extracellular matrix
GPTMS: 3-Glycidoxypropyltrimethoxysilane
HMSN: Hollow mesoporous silica nanoparticle
IκB: Inhibitor of NF-κB
IKK: Inhibitor of nuclear factor kappa-B kinase
KOA: Knee osteoarthritis
MIA: Monosodium iodoacetate
MMP: Matrix metalloproteinases
NF-κB: Nuclear factor kappa-B
OA: Osteoarthritis
PWT: Paw withdrawal threshold
sSiO_2_: Solid SiO_2_
TEOS: Tetraethyl orthosilicate
