# Supplementary material for: Intra-articular delivery of celastrol by hollow mesoporous silica nanoparticles for pH-sensitive anti-inflammatory therapy against knee osteoarthritis
Source: J Nanobiotechnology. 2020 Jul 8;18:94. doi: 10.1186/s12951-020-00651-0 (PMC7346635; doi:10.1186/s12951-020-00651-0)
Supplement: Supplementary file 1 — Additional file 1: S1. Standard curves of CSL/EtOU solution. S2. Time stability of CHC, tested with Fourier transform infrared (FT-IR) spectra 3 months after the nanocomplex was synthesized. S3. Thermogravimetric Analysis (TGA, black curve) and differential scanning calorimetry (DSC, blue curve). S4. Cytotoxicity assay of different concentration (0, 50, 100, 200, 400 μg/mL) after 3 h. There was no significance among each groups. (n = 3, p > 0.05). S5. Paw withdrawal threshold (PWT) of rats treated with saline, 50 mg HMSNs, 100 mg HMSNs, total volume 50 μL. S6. Haematoxylin and eosin (H&E) stained images of hepatic tissue 8 weeks after administration of saline, 50 mg HMSNs, 100 mg HMSNs. S7. Haematoxylin and eosin (H&E) stained images of nephridial tissue 8 weeks after administration of saline, 50 mg HMSNs, 100 mg HMSNs. S8. Haematoxylin and eosin (H&E) stained images of knee joint 8 weeks after administration of saline, 50 mg HMSNs, 100 mg HMSNs. [file 12951_2020_651_MOESM1_ESM.pdf]

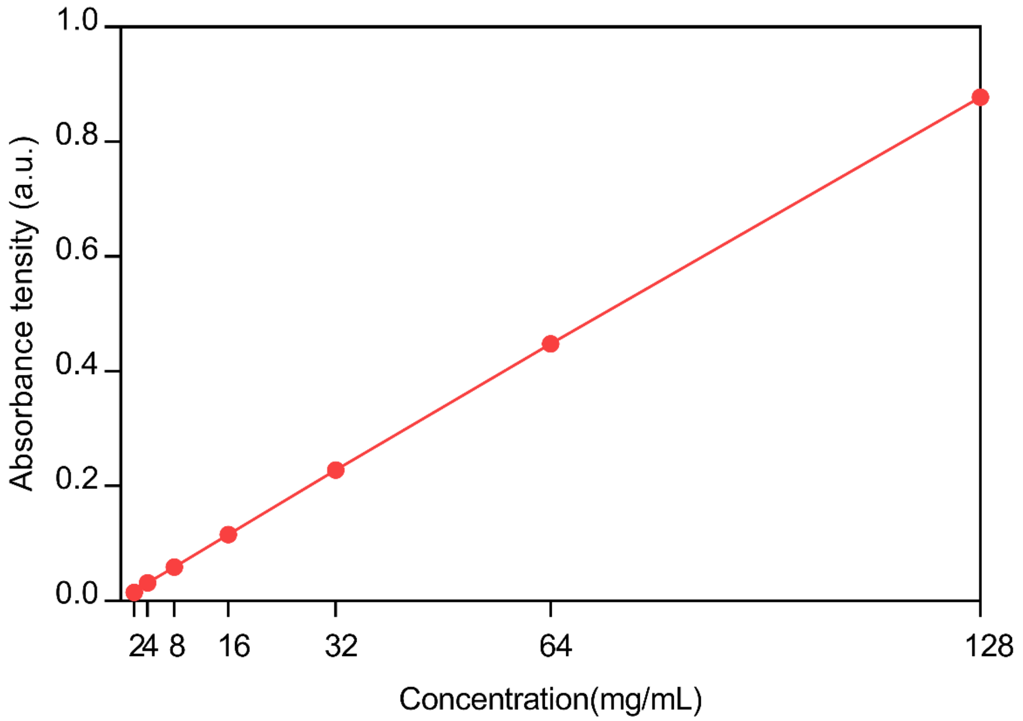

S1. Standard curves of CSL/EtOH solution. y = 0.006846 x + 0.005094, R^2 = 0.999896

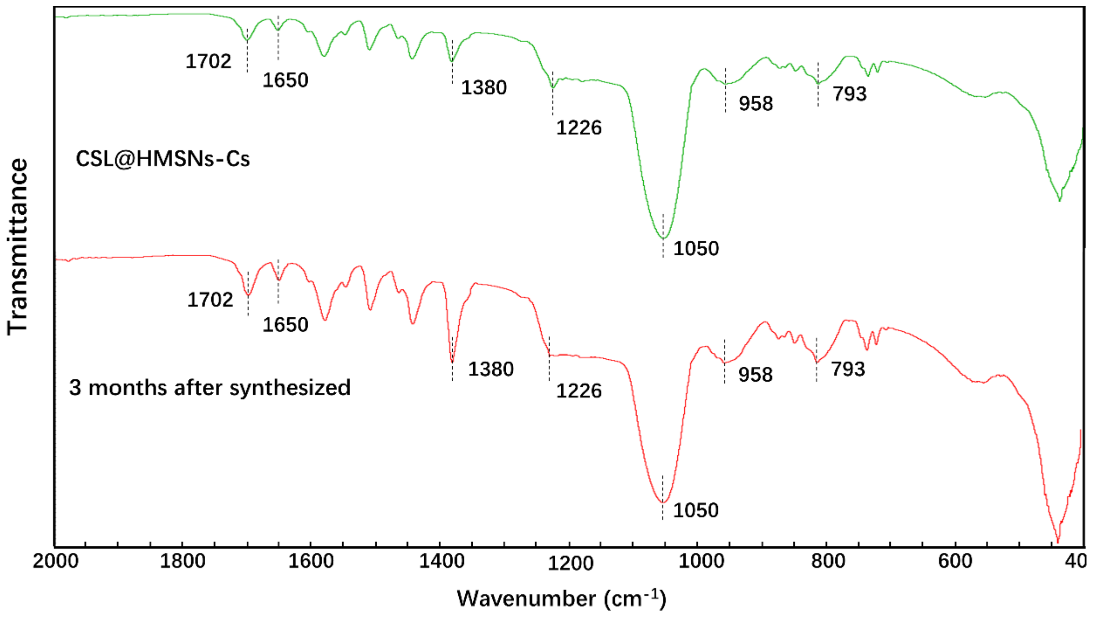

S2. Time stability of CHC, tested with Fourier transform infrared (FT-IR) spectra 3 months after the nanocomplex was synthesized.

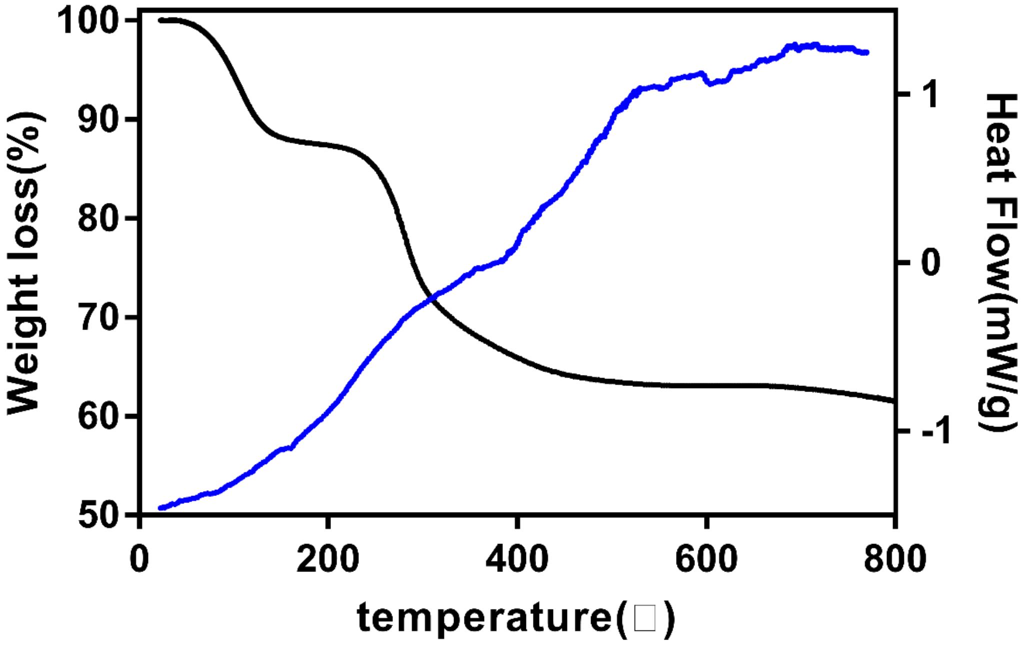

S3. Thermogravimetric Analysis (TGA, black curve) and differential scanning calorimetry (DSC, blue curve).

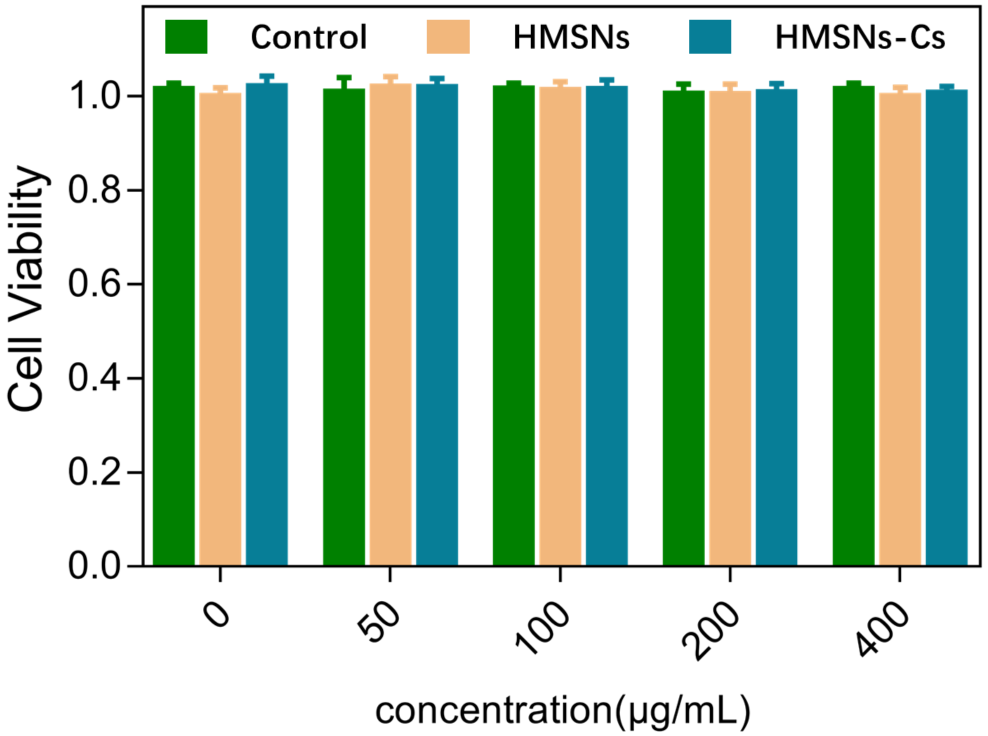

S4. Cytotoxicity assay of different concentration (0, 50, 100, 200, 400μg/mL) after 3 h. There was no significance among each groups. (n=3, $p > 0.05$)

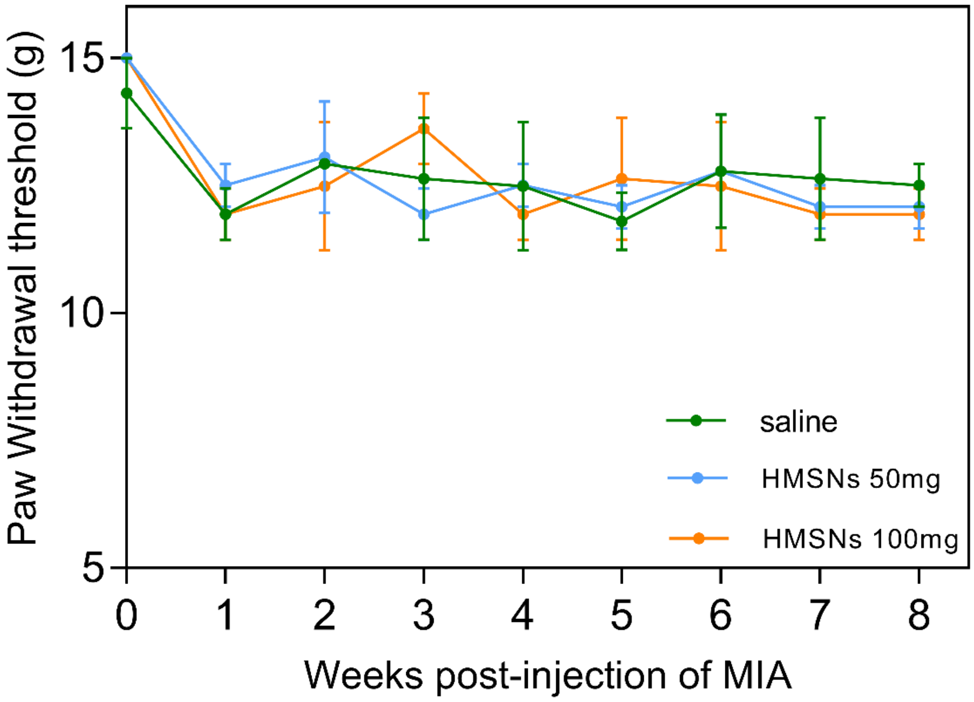

S5. Paw withdrawal threshold(PWT) of rats treated with saline, 50 mg HMSNs, 100 mg HMSNs, total volume 50 µL. There was no significance among each groups. (n=3, $p > 0.05$)

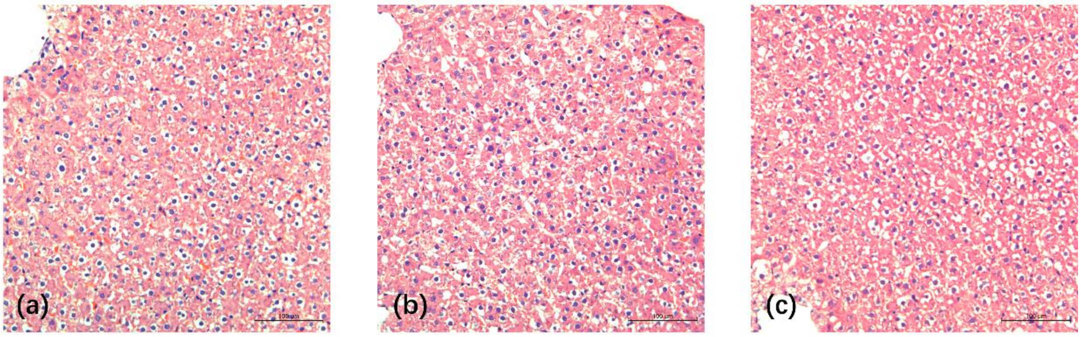

S6. Haematoxylin and eosin (H&E) stained images of hepatic tissue 8 weeks after administration of saline, 50 mg HMSNs, 100 mg HMSNs. There was no significance among each groups.

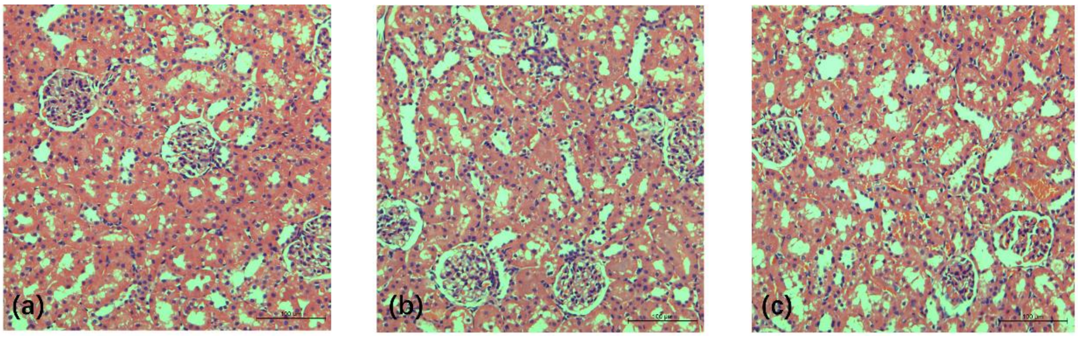

S7. Haematoxylin and eosin (H&E) stained images of nephridial tissue 8 weeks after administration

of saline, 50 mg HMSNs, 100 mg HMSNs. There was no significance among each groups.

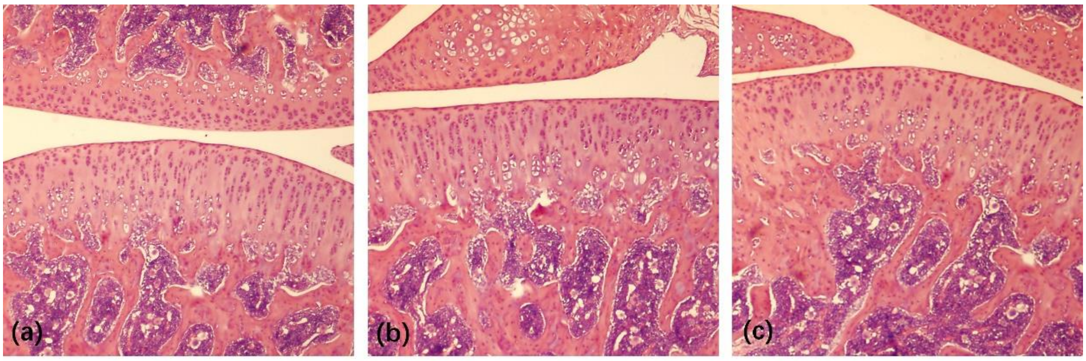

S8. Haematoxylin and eosin (H&E) stained images of knee joint 8 weeks after administration of saline, 50 mg HMSNs, 100 mg HMSNs. There was no significance among each groups.
